# Determination of *ω*-end functionalities in tailored poly(2-alkyl-2-oxazoline)s by liquid chromatography and mass spectrometry

**DOI:** 10.1098/rsos.231008

**Published:** 2024-02-07

**Authors:** Nora Engel, Michael Dirauf, Justyna A. Czaplewska, Ivo Nischang, Michael Gottschaldt, Ulrich S. Schubert

**Affiliations:** ^1^ Laboratory of Organic and Macromolecular Chemistry (IOMC), Friedrich Schiller University Jena, Humboldtstraße 10, 07743 Jena, Germany; ^2^ Jena Center for Soft Matter (JCSM), Friedrich Schiller University Jena, Philosophenweg 7, 07743 Jena, Germany; ^3^ NGP Polymers GmbH, Philosophenweg 7a, 07743 Jena; ^4^ Helmholtz-Zentrum Berlin für Materialien und Energie GmbH (HZB), Hahn-Meitner-Platz 1, 14109 Berlin, Germany; ^5^ Helmholtz Institute for Polymers in Energy Applications Jena (HIPOLE Jena), Lessingstraße 12–14, 07743 Jena, Germany

**Keywords:** cationic ring-opening polymerization (CROP), liquid chromatography, poly(2-oxazoline)s (POx), matrix-assisted laser desorption/ionization time-of-flight mass spectrometry (MALDI-TOF MS), electrospray ionization mass spectrometry (ESI MS)

## Abstract

The in-depth analytical characterization of polymers, in particular regarding intended biomedical applications, is becoming increasingly important to elucidate their structure–property relationships. Specifically, end group analysis of e.g. polymers featuring a ‘stealth effect’ towards the immune system is of particular importance because of their use in coupling reactions to bioactive compounds. Herein, we established a liquid chromatography (LC) protocol to analyse bicyclo[6.1.0]nonyne-functionalized poly(2-alkyl-2–oxazoline)s (POx)s as promising functional polymers that can be applied in strain-promoted click reactions. This work involved the synthesis of poly(2-methyl-2-oxazoline) (PMeOx) and poly(2-ethyl-2-oxazoline) (PEtOx) by living cationic ring-opening polymerization (CROP) with different molar masses ranging from 2 up to 17.5 kDa and, to our knowledge, the first liquid chromatographic analysis of PMeOx. The developed analytical protocol enables the quantitative determination of post-polymerization reaction sequences with respect to the conversion of the *ω*-end groups. All synthesized polymers were straightforwardly analysed on a C18-derivatized silica monolithic column under reversed-phase chromatographic conditions with a binary mobile phase gradient comprising a mixture of acetonitrile and water. Subsequent mass spectrometry of collected elution fractions enabled the confirmation of the desired *ω*-end group functionalities and the identification of synthetic by-products.

## 1. Introduction

Research on polymer–drug conjugates in the pharmaceutical industry is distinctly marked by a growing interest and has to meet defined good manufacturing practice (GMP) guidelines, implied, e.g. by the European Medicines Agency (EMA) or the Food and Drug Administration (FDA) [1,2]. In order to receive an official approval later, the polymers have to be well-defined by means of dispersity indices, molar masses and their respective distinct end groups. Poly(ethylene glycol)s (PEGs) are already established and approved by the FDA and EMA for conjugation to a wide range of drugs by a so-called PEGylation reaction. A significant drawback of the broad use of PEGs are recent reports of antibodies identified in humans. Those can impose a risk and reduce the efficacy of PEG-based drug delivery systems [3–5]. Discussed alternatives to PEG include, e.g. polyphosphates [6,7], polyglycerols [8], polypeptides [9], and also hydrophilic poly(2-alkyl-2-oxazoline)s (POx) [10–12].

Since the first reports on their synthesis in the 1960s [13–16], POx represent an emerging class of polymers for bioconjugation reactions and are, therefore, discussed as an alternative to today's gold standard pharma polymer PEG. The versatility of the synthesis of POx enables the introduction of a broad range of functionalities addressing different application purposes [17]. The utilization of water-soluble POx for biomedical applications, also by introduction of tailor-made functionalities within the polymer structure, enables well-controlled coupling reactions with bioactive compounds [18,19]. POxylated rotigotine (around 20 kDa) from Serina Therapeutics Inc. (Huntsville, USA), already in clinical development for the treatment of early stage Parkinson [20]. Avroxa Polymers (Ghent, Belgium), is trading various types of POx with molar masses from 5 to 50 kDa under the brand name Ultroxa^®^ [21].

Due to this growing interest in POx, a detailed analysis and quantification of occurring *α*- and *ω*-end groups for, e.g. subsequent click reactions, is in focus of research and development. Side reactions of the cationic ring-opening polymerization (CROP) of POx include, among others, chain-transfer to monomer by *β*-elimination or chain transfer to a cationically charged enamine chain end [22]. Those can result in a varying proportion of hydrogen-initiated polymer chains [23]. An in-depth investigation of the side reactions occurring during initiation and polymerization with a focus on the *α*-end group has already been carried out. The study enabled identification and quantification of hydrogen-initiated polymer chains as well as other chain transfer products [24].

In this study, we mainly focus on the characterization of hydrophilic POx with respect to their *ω*-end group functionalities obtained from coupling reactions through a two-step post-polymerization modification reaction. Typical functional groups for subsequent click reactions are e.g. carboxylic acids [25,26], maleimide [27,28], dibenzocyclooctyne (DBCO) [29–31], or bicyclo[6.1.0]nonyne (BCN) [29,30,32]. The resultant POx-protein conjugates have already been reported and were enabled by using different functional groups [33]. However, from an analytical perspective, knowledge of the degree of functionalization (DF) by suitable analytics can be very desirable for achieving quantitative conversion in site-specific conjugation reactions [34,35].

The DF of most *ω*-end groups of POx can be determined by proton nuclear magnetic resonance (^1^H NMR) spectroscopy. However, this method already reaches its limit and quantitative meaning in regard to the end group for POx with molar masses exceeding 10 kDa, due to the increasingly low signal-to-noise ratios. Matrix-assisted laser desorption/ionization (MALDI) or electron spray ionization (ESI) mass spectrometry (MS) can be applied in order to identify polymers with the desired end groups, but these techniques do not provide quantitative values because of the well-known mass discrimination effects [36,37]. Due to the used ionization method, ESI MS is only applicable for POx with lower degree of polymerization (DP) values, in part due to the occurrence of multiple-charged species in *m/z* distributions, particularly with increasing overall chain lengths. Using MALDI time-of-flight (MALDI-TOF) MS, the ionization of polymers with higher molar mass, e.g. 15 kDa is possible but limited to the repeating unit, dispersity as well as the purity of the polymer [37]. For low molar mass POx (DP up to 20), the identification and quantification of *α*-end groups was possible by applying liquid chromatography (LC) with subsequent ESI MS. First side reactions were already identified during the polymerization process of the 2-alkyl-2-oxazoline monomer [23,24]. However, hydrogen-initiated species as well as hydroxy-terminated polymer species could be identified and quantified with the developed analytical protocol [24].

Here, we present a LC protocol to separate hydrophilic POx featuring different *ω*-end groups that originate from established synthetic reaction pathways [29,38]. Particularly promising hydrophilic POx, poly(2-methyl-2-oxazoline)s (PMeOx) and poly(2-ethyl-2-oxazoline)s (PEtOx), as potential alternative ‘stealth polymers’ to substitute PEG in the areas of drug conjugation and for formulations, were chosen. Next to their use in purely research-focused studies, selected examples are already successfully applied, e.g. POxylated rotigotine [20]. Both, hydrophilic PEtOx and PMeOx of varying DPs are promising candidates for such applications that only differ in the length of the alkyl substituent modulating hydrophilicity probably also affecting the stealth properties of drug conjugates and formulations. To elucidate the structure–property relationships and to use the respective materials in envisaged applications, we aimed for investigations of POx of varying DPs, ranging from 20 to 175. We, thereby, unveil the potential of properly established LC protocols extending the toolbox for a detailed characterization and informed use of these polymers in future formulation and bioconjugation reactions.

## 2. Results and discussion

**Scheme 1.**
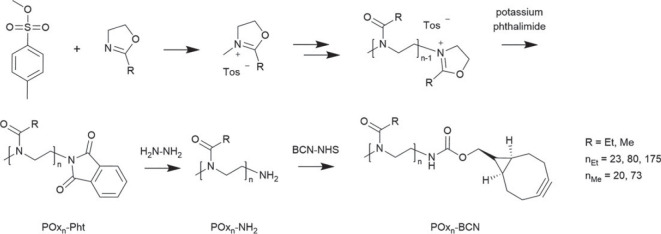
Schematic representation of the mechanism of the CROP (top) using 2-ethyl- or 2-methyl-2-oxazoline (R = Et, Me) as monomers and potassium phthalimide as terminating agent. Post-polymerization modification reactions (bottom) start with the hydrazinolysis of the phthalimide-functionalized POx (POx_n_-Pht) to the corresponding amino group bearing POx (POx_n_-NH_2_) followed by the reaction with BCN-NHS leading to the BCN-functionalized POx (POx_n_-BCN).

The CROP of either 2-ethyl-2-oxazoline or 2-methyl-2-oxazoline was initiated by methyl *p*-tosylate (MeTos) (scheme 1). It was reported in the literature that phthalimide moieties could be introduced via a Mitsunobu reaction of the hydroxyl terminal POx resulting in a degree of functionalization (DF) for poly(2-isopropyl-2-oxazoline) (P*iPr*POx) of around 70% [39]. We decided to directly terminate the polymerization reaction by the addition of potassium phthalimide to the reactive polymer chains in order to reduce the number of synthetic steps and to obtain higher DF values [40]. Subsequent hydrazinolysis of the phthalimide moieties led to the respective amino *ω*-end group bearing polymers. Reaction with (1*R*,8*S*,9*s*)–bicyclo[6.1.0]non-4-in-9-ylmethyl]-*N*-succinimidylcarbonat (BCN-NHS) resulted in bicyclo[6.1.0]nonyne carrying POx suitable for strain-promoted copper-free click reactions, e.g. with Interferon-*α*2A [29,32,38]. The reaction sequence was applied for PEtOx with a DP of 23, 80, and 175 as well as for PMeOx with a DP of 20 and 73. All polymers in scheme 1 were first analysed by ^1^H NMR and size-exclusion chromatography (SEC) and exhibited molar masses in the desired range (see electronic supplementary material, §S2, figures S1 to S15 and table S1).

For polymers which could be ionized, the DP as well as the end groups were analysed by ESI or MALDI-TOF MS and the corresponding DF was determined by suitable ^1^H NMR signals if applicable (see electronic supplementary material, §S1, figures S1 to S9 and S11 to S16). Due to low signal-to-noise ratios concerning the PEtOx_175_ series it was challenging to receive accurate results. We performed ^1^H NMR measurements of PEtOx_175_-Pht at 24°C and 50°C as well as two different number of transients (NT = 16 or 256) in order to increase the resolution of the ^1^H NMR spectrum (see electronic supplementary material, §S1, figure S10) but it did not result in any improvement. However, for the PEtOx_23_-Pht, the SEC trace revealed a dispersity of 1.13 and a number-average molar mass (M_n_) of 4.2 kg mol^−1^ (see electronic supplementary material, §S2.1, figure S1). The end groups were confirmed by MALDI-TOF MS. The DF, which was determined through integration of suitable ^1^H NMR signals, namely the signals originating from the aromatic ring system of the phthalimide at 7.9 ppm as well as the polymer backbone signal at around 3.45 ppm, was found to be quantitative.

**Figure 1.**
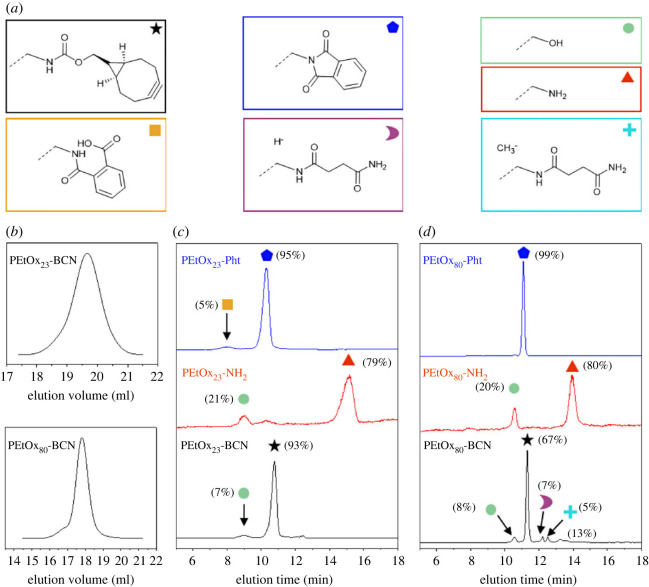
(*a*) Schematic representation of all end groups in polymer species, (*b*) SEC traces of the desired PEtOx_n_-BCN (*n* = 23, 80) and comparison of elugrams obtained from separations of *ω*-end group functionalized PEtOx_n_-BCN/NH_2_/Pht of (*c*) PEtOx_23_ and (*d*) PEtOx_80_ with phthalimide (blue line), amino (red line) and BCN (black line) moieties including assignment of the correspondingly separated polymer species identified by ESI or MALDI-TOF MS data (see electronic supplementary material, §S5.1, figures S18 to S21).

In contrast to the SEC distribution of PEtOx_23_-Pht, the respective HPLC elugram revealed a second peak population (electronic supplementary material, figure S1C). The elution fractions were collected and analysed by ESI MS. Analysis of the first fraction (collected between 6.3 and 8.4 min) revealed a product originating from a partial hydrolysis of the phthalimide (see electronic supplementary material, §S3, figure S17). After hydrazinolysis to PEtOx_23_-NH_2_ (figure 1*c*, red line), two major peak populations were observed. Based on the ESI MS data of the collected elution fractions (see electronic supplementary material, §S5.1, figure S18), the first fraction at 8.97 min was assigned to the hydroxy-end functionalized polymer and the elution population at 14.51 min was assigned to the desired PEtOx_23_-NH_2_. The polymer population at 10.1 min could not be analysed by mass spectrometry due to the low concentration. Based on the location in the elution trace, it is remaining non-hydrolysed phthalimide. However, the signal originating from partially hydrolysed phthalimide disappeared after hydrazinolysis because it is also converted to an amino moiety [41].

**Figure 2.**
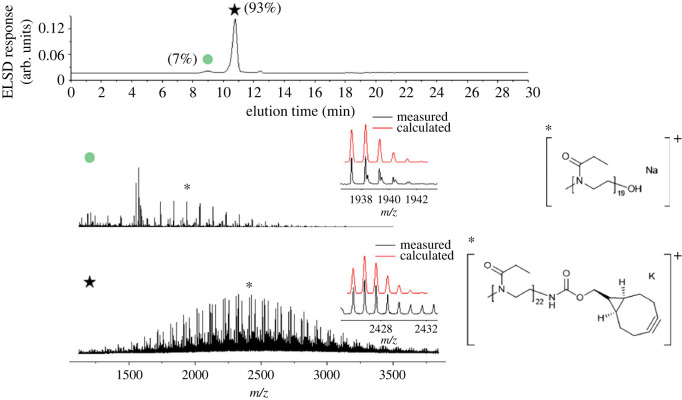
Elugram of PEtOx_23_-BCN and MALDI-TOF MS (first fraction measured in DCTB and NaTFA, second fraction measured in DHB) of the collected fractions indicated in the HPLC elugram with assignment of marked *m/z* polymer species. ELSD: evaporative light scattering detector.

In the next step, PEtOx_23_-NH_2_ was reacted with BCN-NHS to attach the bicyclo[6.1.0]nonyne functionality to the *ω*-end of the polymer chain. The SEC trace (figure 1*b*, PEtOx_23_-BCN) of the product revealed a mass distribution with a dispersity of Ð = 1.13. In the correspondingly recorded HPLC elugram, the hydroxy-terminated species (9.06 min) as well as the desired BCN-functionalized species (10.77 min) were separated and identified (figure 2). It should be noted that instead of the desired polymers with carbamate-bound BCN groups also a certain amount of polymer with carbonate-bound BCN groups might have been formed from the previously detected PEtOx_23_-OH (since also the DF increases from 79% of amine substituted polymer to 93% for the polymer with a BCN end group). It cannot be excluded that the polymer species with BCN bound via carbonate functionality co-elutes with the desired PEtOx_23_-BCN. However, these results demonstrate that with the protocol, different polymer species can be identified in the reaction sequence of PEtOx with a molar mass centring around 2 kDa.

To investigate the suitability of the chromatographic protocol, PEtOx with higher molar masses, centring around 8 kDa, with the very same end groups (PEtOx_80_-Pht/NH_2_/BCN) were synthesized. For the polymers in this molar mass range also monomodal SEC traces were recorded, except that a shoulder at lower elution volumes appeared visible (figure 1*b*, PEtOx_80_-BCN). End groups were identified by MALDI-TOF MS by evaporating the solvent prior to the sample preparation and measurement. Quantification of the DF by ^1^H NMR was limited due to the sensitivity of the NMR instrument. Due to low signal-to-noise ratios concerning the PEtOx_175_ series it was challenging to receive accurate results. ^1^H NMR measurements of PEtOx_175_-Pht at 24°C and 50°C with two different numbers of transients (NT = 16 or 256), in order to increase the resolution of the ^1^H NMR spectra (see electronic supplementary material, §S1, figures S10), did not result in any significant improvement. In the HPLC elugram of PEtOx_80_-Pht (figure 1*d*, blue line), the first signal at 10.55 min was assigned to the hydroxy-terminated PEtOx_80_ applying MALDI-TOF MS of the collected fraction (see electronic supplementary material, §S5.1, figure S18). By integration of the peak area (*n* = 1), a DF of 99% was calculated (compared with DF_NMR_ = 90%).

In contrast to PEtOx_23_-Pht, the partly hydrolysed phthalimide could not be identified in the initial HPLC measurement of the freshly prepared sample. Twelve days of polymer storage in the initial eluent mixture (90/10 water/acetonitrile, % (v/v)) resulted in the appearance of the partially hydrolysed phthalimide at an elution time of 9.01 min (see electronic supplementary material, §S5.1, figure S20) as well.

The elugram of the PEtOx_80_-NH_2_ (figure 1*d*, red line) has a similar pattern as the PEtOx_23_-NH_2_ and comprises two different populations. The signal with lower abundance at 10.55 min originates from PEtOx_80_-OH (see electronic supplementary material, §S5.1, figure S21). The broad population at an elution time of 13.94 min stems from the desired PEtOx_80_-NH_2_. The DF of PEtOx_80_-NH_2_ determined by the integration area is 80% (*n* = 1), indicating 20% of the hydroxyl-terminated by-product, and is similar to that for the PEtOx_23_-NH_2_.

For PEtOx_80_-BCN, the SEC trace (figure 1*b*) exhibited a high molar mass shoulder as mentioned before; however, MALDI-TOF MS confirmed the successful attachment of the BCN functionality (see electronic supplementary material, §S2.1, figure S6). ^1^H NMR could not be used for the determination of the DF due to the low signal-to-noise ratio (see electronic supplementary material, §S2.1, figure S6B).

A serial dilution of PEtOx_80_-BCN (see electronic supplementary material, §S5.2, figure S23) indicated linearity of the used evaporative light scattering detector (ELSD) in the investigated concentration range in a double logarithmic plot. This enabled quantification of the DF of PEtOx_80_-BCN, which was 95% ± 0.34% (*n* = 5) (see electronic supplementary material, §S4.2, figure S24A). In order to identify the different peak populations, we remeasured the same polymer after more than one year. In the elugram (figure 1*d*, black line) a DF of 67% was determined, clearly demonstrating a decrease in the DF, which is not unexpected for storing a sample in solution over more than one year.

However, in the HPLC elugram (figure 1*d*, black line), five separated peaks were recorded. An assignment of almost all signals was possible after separation and MALDI-TOF MS (see electronic supplementary material, §S5.1, figure S21). The first signal was attributed to the hydroxy-end functionalized PEtOx_80_ and the second elution fraction at 11.31 min to the desired PEtOx_80_-BCN. It should be noted that instead of the desired carbamate also carbonates could have been formed that could co-elute (as already described for PEtOx_23_-BCN). Apart from those two populations, the elugram of PEtOx_80_-BCN revealed two additional populations at higher elution times. Those were assigned to side products originating from the NHS-ester coupling reactions. The established protocol is additionally capable to distinguish between the hydrogen-initiated side product which eluted earlier compared with the methyl-initiated one, as observed also for other POx [24].

In order to elucidate the limits of the applied separation protocol, PEtOx with a DP of 175 was synthesized following the identical reaction sequence as depicted in scheme 1. For the polymers in this molar mass range, the analysis protocol reached its limits. It was not possible to identify baseline separated signals in the elugrams (see electronic supplementary material, §S5.2, figure S25).

**Figure 3.**
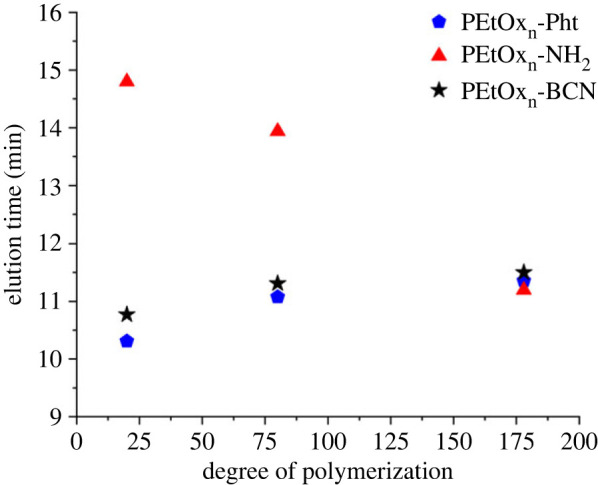
Dependence of elution time on the DP of PEtOx_n_ end-functionalized with phthalimide (-Pht, blue), amino (-NH_2_, red), and bicyclo[6.1.0]nonyne moieties (-BCN, black).

Based on these results for the series of PEtOx_n_, the dependence of the elution time on varying average DPs of polymers for the different end groups are summarized in figure 3. With increasing DP, the elution times of the PEtOx_n_-Pht (figure 3, blue) increased.

PEtOx_n_-BCN eluted similar to the phthalimide-functionalized species with slightly increased elution times, particularly visible for the lower DPs. A somewhat reverse effect was monitored for PEtOx_n_-NH_2_. The elution times changed evidently to much lower values at increased DPs, reaching similar values of approximately 11.5 min as the other functionalities at a DP of 175. The influence of the *ω*-end group's functionality on the elution behaviour decreases with increasing chain length, which is expected [42]. This limits the applicability of the presently selected elution conditions for distinguishing end groups at higher DPs.

**Figure 4.**
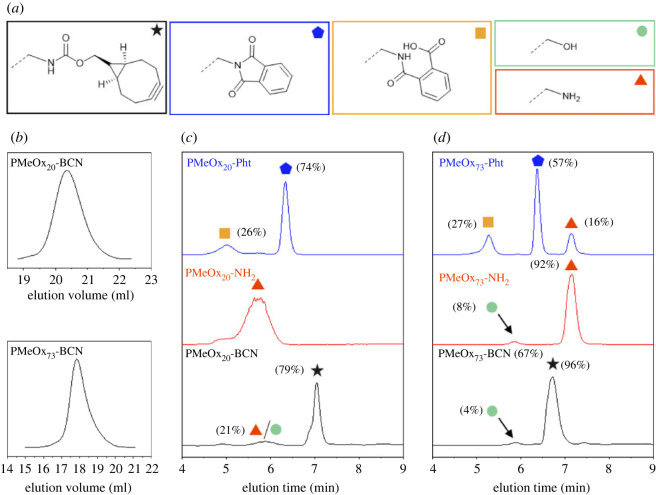
(*a*) Schematic representation of all identified polymer species, (*b*) SEC traces of the desired PMeOx_n_-BCN (*n* = 20 and 73) and comparison of elugrams of the reaction sequence products with the *ω*-end group functionalized PMeOx_n_-Pht/NH_2_/BCN of (*c*) PMeOx_20_ and (*d*) PMeOx_73_ with phthalimide (blue line), amino (red line) and BCN (black line) moieties. As well included are assignment of the corresponding polymer species by comparison with PEtOx_n_ elugrams.

Another, even more hydrophilic POx, PMeOx_n_-Pht/NH_2_/BCN was synthesized and investigated as well (scheme 1), despite difficulties for analysis by ESI and MALDI-TOF MS in most instances. For the synthesized polymers, SEC traces indicate monodisperse populations with dispersity values of Ð < 1.2. The calculation of the DF was done—if applicable—as for PEtOx_n_, by integration of the suitable signals from ^1^H NMR measurements (see electronic supplementary material, §S4, table S1). HPLC elugrams of all PMeOx_n_ bearing –Pht/NH_2_/BCN as *ω*-end groups eluted at lower retention times compared with PEtOx_n_ (figure 4), indicating a significantly higher hydrophilicity of the former. The analysis by MS was only possible for PMeOx_23_-Pht (see electronic supplementary material, §S2.2, figure S10) but not for other PMeOx even from the collected elution populations. The assignment of polymeric species was, therefore, deduced from the comparison with the elugram pattern of PEtOx_n_.

**Figure 5.**
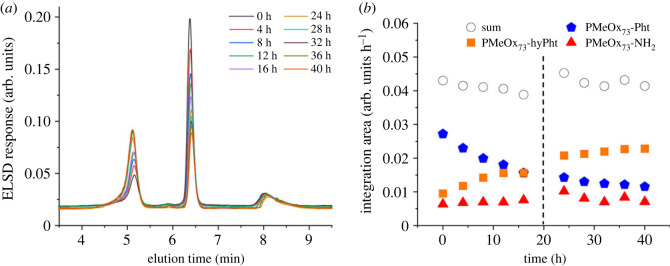
(*a*) Elugrams of PMeOx_73_-Pht dissolved in the start eluent (90/10 water/acetonitrile, % (v/v)) at the selected time points and (*b*) development of elution abundance of each species and their sum over the timescale of investigation.

For PMeOx_20_-Pht, the signal assignment was executed by comparison with the elugram of PEtOx_23_-Pht. The first signal was identified to be the product from the partial hydrolysis of the phthalimide-end group (figure 4*c*, blue line). By remeasurement of the same sample stored in the eluent mixture (90/10 water/acetonitrile, % (v/v)), the same phenomenon was observed as shown in figure 1 for PEtOx_23_-Pht. However, for PMeOx_20_-NH_2_ (figure 6*c*, red line), the elugram pattern was slightly different compared with PEtOx_23_-NH_2_. No clear baseline-separated peak populations were observed. Both the hydroxy-terminated as well as the desired PMeOx_20_-NH_2_ species, could co-elute under the present chromatographic conditions and it was not possible to determine the DF based on the integration area. The elugram of PMeOx_20_-BCN (figure 5*c*, black line) was similar to the PEtOx_23_-BCN. However, the first population at elution times of *ca* 5.90 min could originate from the remaining PMeOx_20_-NH_2_ or the PMeOx_20_-OH species or PMeOx_73_-Pht (figure 4*d*, blue line) since three baseline-separated signals were observable. The prominent signal at 5.27 min was identified to be the polymer with a partially hydrolysed phthalimide-end group and the second population at 6.37 min to be the PMeOx_73_-Pht as the desired product. The third signal at 7.15 min could be assigned to the PMeOx_73_-NH_2_ population by comparison of the elution time with PMeOx_73_-NH_2_ (figure 4*d*, red line).

Comparing all POx_n_-Pht, the partially hydrolysed species from PMeOx_73_-Pht was very prominent already in the initial measurement. Remeasurement of the same sample stored in the eluent mixture (90/10 water/acetonitrile, % (v/v)) over 40 h revealed that the population of PMeOx_73_-hyPht at 4.9 min increased in abundance while the signal originating from phthalimide at 6.2 min decreased in abundance (figure 5*a*).

The sum of integration areas, including all populations, as well as the integration area for PMeOx_73_-NH_2_ remained almost invariant over the timescale of the investigation (figure 5*b*). The shift in the trend of the data (figure 5*b*, indicated by the dotted line) resulted from an intermediate shutdown of the system, in particular the ELSD, before the measurement series was re-assumed. For PMeOx_73_-hyPht and PMeOx_73_-Pht, a trend plateau is indicated after 40 h of investigation.

The partial hydrolysis of PEtOx_80_-Pht was slower when compared with PMeOx_73_-Pht (comparing electronic supplementary material, figure S22 in §S5.2 and figure 5). Although it is known that the ELSD response correlates with the DP of the corresponding polymers, in our case only the end group differs [43]. The other, though small, population in the elugram of PMeOx_73_-NH_2_ at 5.88 min was assigned to PMeOx_73_-OH in comparison with the corresponding elugram of PEtOx_80_.

Remeasurements of the amino-containing PMeOx_73_ over a timescale of 10 days resulted in significant shifts of elution times with some reduction in abundance of the population (see electronic supplementary material, §S5.2, figure S26). The time of storage of the sample in an aqueous solution appears to have an influence on the experimental results. We observed a delayed elution and broadening of the population. This phenomenon might be due to the protonation of the amino moiety of POx_n_-NH_2_. Therefore, freshly prepared samples have to be used for the investigation and applied in subsequent reactions in order to be quantitative. However, from the measurement of the freshly prepared sample from PMeOx_73_-NH_2_, a DF of 96% was calculated, which is higher when compared with the PEtOx_n_-NH_2_. For PMeOx_73_-BCN two prominent populations were recorded (figure 5*d*, black line). Most probably, the hydroxy- and the amino-terminated species were co-eluting. The main population at 6.08 min elution time was allocated to the BCN-functionalized polymer. The DF determined by integration of the peak area in the HPLC elugram was 96% ± 0.55% (*n* = 5) (see electronic supplementary material, §S5.2, figure S24B).

## 3. Conclusion

Two series of hydrophilic poly(2-alkyl-2-oxazoline)s with polymerization degrees from 20 up to 175, functionalized with phthalimide, amino and bicyclo[6.1.0]nonyne moieties were synthesized and analysed by an optimized liquid chromatography protocol with subsequent mass spectrometry of the collected elution fractions. The established liquid chromatography protocol comprises a gradient elution on a monolithic, C18-modified silica column coupled to an evaporative light scattering detector to separate and identify the desired products as well as the undesired by-products in the synthetic protocols. Particular focus was on the *ω*-end groups from defined post-polymerization reaction sequences of potential interest for later POxylation reactions. The separated and collected elution populations seen in the elugrams were identified and investigated by subsequent ESI and MALDI-TOF MS investigations. Detailed analysis of the stored samples over time revealed also a partial conversion, in particular, of the phthalimide bearing end groups. The protocol demonstrates the possibility to further track and quantify post-polymerization modifications in addition to commonly used methods and is believed to be of pivotal importance to optimize and tailor product homogeneity and outcome in bio-orthogonal POxylation reactions.

## 4. Method

### 4.1. Materials

Barium oxide (BaO, 90%, ACROS), calcium hydride (CaH_2_, Sigma-Aldrich), triethylamine (TEA, Sigma-Aldrich), potassium phthalimide (Sigma-Aldrich), hydrazine monohydrate (N_2_H_2_, 64 to 66% N_2_H_4_, Sigma-Aldrich), sodium trifluoroacetate (NaTFA, 98%, Sigma-Aldrich), 2,5-dihydroxybenzoic acid (DHB, 98%, Sigma-Aldrich) were used as purchased. Prior to use, 2-ethyl-2-oxazoline (EtOx, Sigma-Aldrich) and 2-methyl-2-oxazoline (MeOx, Sigma-Aldrich) were stirred over BaO overnight, distilled and stored under argon atmosphere. Methyl *p*-tosylate (MeTos, Sigma-Aldrich) was pre-dried over CaH_2_, distilled under reduced pressure and stored under argon atmosphere. Acetonitrile was obtained from a solvent purification system (SPS; Pure solv EN, InnovativeTechnology). HPLC and MS grade acetonitrile and water for HPLC measurements were obtained from VWR. All other chemicals and solvents were received from common commercial sources and used without further purification unless otherwise stated.

### 4.2. Instrumentation

Nuclear magnetic resonance (NMR) spectra were recorded at room temperature on a 300 MHz spectrometer from Bruker equipped with an Avance I console, a dual proton (^1^H) and carbon (^13^C) sample head and a 120 × BACS automatic sample changer in CDCl_3_ or CD_2_Cl_2_. All chemical shifts are given in ppm and were determined using the residual non-deuterated solvent signal as the reference.

Size exclusion chromatography (SEC) was performed on an Aglient 1200 series, equipped with a PSS degasser, a G1310A pump, a G1329A auto sampler, a Techlab oven (40°C) and a G7162A refractive index detector for data acquisition. As eluent, a mixture of DMAc and 0.21 wt% LiCl with a flow rate of 1 ml min^−1^ was used. A PSS GRAM guard/30/1000 Å (10 µm particle size) was used as the column. Molar masses were estimated using polystyrene (PS) or poly(ethylene glycol) (PEG) polymers as standards (*ca* 400 to 1 000 000 g mol^−1^).

Matrix-assisted laser desorption/ionization time-of-flight mass spectrometry (MALDI-TOF MS) measurements were carried out on a rapifleX MALDI-TOF/TOF System (Bruker Daltonics) equipped with a scoutMTP II ion source and a smartbeam™ 3D laser (355 nm wavelength). All spectra were acquired in positive reflector mode using *trans*-2-[3-(4-tert-butylphenyl)-2-methyl-2-propenylidene]malononitrile (DCTB) with sodium trifluoroacetate (NaTFA) or 2,4-dihydroxybenzoic acid (DHB) as the matrix. General sample preparation: 1–5 mg of the polymer was dissolved in 50–200 µl CHCl_3_; 0.40 µl of a pre-mixed solution containing 20 µl of DCTB (20 mg ml^–1^ dissolved in CHCl_3_) with 2.5 µl of NaTFA (2 mg ml^–1^ dissolved in THF) or DHB (30 mg ml^−1^) and 10 µl polymer solution was deposited on a MTP target frame III ground steel plate. The recording was performed using the manufacturer's software flexControl 4.0 with individual setting adjustments (e.g. laser energy) resulting in a large range of recorded single spectra for different samples. The evaluation and processing of the recorded spectra were accomplished using the manufacturer's software flexAnalysis 4.0 including baseline subtraction and external calibration by different PMMA (2500, 5000 and 10 000 Da) standards as calibrants.

Electrospray ionization mass spectrometry (ESI MS) was carried out on a Bruker MicrQTOF mass spectrometer equipped with an ESI source using a calibration standard ‘ESI-L Low Concentration Tuning Mix’ supplied by Agilent.

Lyophilization of polymers was conducted on an Alpha 1 2 LD plus freeze dryer from Martin Christ Gefriertrocknungsanlagen GmbH (Osterode am Harz, Germany).

On an initiator single-mode microwave synthesizer from Biotage (Uppsala, Sweden), equipped with a non-invasive IR sensor (temperature accuracy: 2%), selected polymerization reactions under microwave irradiation were performed.

High performance liquid chromatography (HPLC) measurements were carried out on an Agilent Technologies 1200 series chromatographic system from Polymer Standards Service GmbH (PSS, Mainz, Germany) comprising a SofTA Model 400 ELSD with nitrogen as the carrier gas for elution monitoring. The spray chamber temperature was set to 45°C, whereas the drift tube temperature was set to 70°C. The detector was operated at the maximum data acquisition rate of 10 Hz. The column was placed in a TCC 6000 column oven from Polymer Standards Service GmbH (PSS, Mainz, Germany) tempered at 30°C. The column was connected to the injector and detector via a 130 µm internal diameter tubing to minimize extra-column band broadening. As the stationary phase, a Chromolith High Resolution RP18 end-capped silica monolithic column from Merck KGaA (Darmstadt, Germany) was used. The column had a nominal length of 100 mm at an internal diameter of 4.6 mm.

The HPLC analysis protocols were developed based on established literature procedures [24]. The sample concentration was 1 or 2 mg ml^–1^ in typical elution experiments. The used gradient elution programming for HPLC measurements comprised a binary mobile phase solvent composition of acetonitrile and water (% (v/v)) with the following elution programming: linear gradient of acetonitrile from 10% to 90% (v/v) within 20 min, followed by an isocratic hold for 10 min. Afterwards, initial elution conditions were reconstituted by a linear decrease of acetonitrile from 90 to 10% within 5 min and an isocratic hold for 1 min. Prior to the next injection, an additional isocratic hold for 15 min was programmed. The injection volume was 10 µl and the flow rate was set to 1 ml min^–1^.

### 4.3. Synthesis of poly(2-ethyl-2-oxazoline)s (PEtOx)

The series of PEtOx_n_-Pht/NH_2_/BCN polymers were synthesized analogously to previously published protocols [24,35,38]. Detailed information concerning the synthesis can be found in the electronic supplementary material, §S1.

### 4.4. Synthesis of poly(2-methyl-2-oxazoline)s (PMeOx)

#### 4.4.1. Synthesis of PMeOx_20_-Pht

A pre-heated two-necked flask with stirrer connected to a Findenser™ with T-piece and a gas bubbler was evacuated thrice, heated up and cooled down under a gentle flow of argon. MeTos (1.81 ml, 2.23 g, 12 mmol, 1 eq) and MeOx (20.82 ml, 20.42 g, 240 mmol, 20 eq) were dissolved in 37 ml acetonitrile, heated to reflux, and stirred for 2 h under a gentle flow of argon. Then, potassium phthalimide (6.00 g, 32 mmol, 2.7 eq) was added and the temperature was set to 70°C. The reaction mixture was stirred overnight, cooled to room temperature, and the precipitate was filtered off. The solvent was removed under reduced pressure. The residue was resuspended in 200 ml distilled CH_2_Cl_2_ and filtered. The solvent was removed under reduced pressure and the residue was dried *in vacuo*. The crude product was redissolved in 150 ml deionized water and dialyzed against deionized water. The polymer solution was freeze-dried and the polymer was obtained as a colourless solid. Yield: 18.2 g (89%). Conversion: 98%. DP_NMR_ = 20. DF_NMR_ = 71%. ^1^H NMR (300 MHz, CDCl_3_): *δ* = 7.40–8.06 (m, 3H), 3.47 (br s, 66H), 3.06 (br s, 3H), 1.82–2.50 (m, 63H), SEC (DMAc, 0.21 wt% LiCl, PS–cal./PEG–cal.): M_n,PS–cal._ = 3.9 kg mol^−1^, M_w,PS–cal._ = 4.3 kg mol^−1^, Đ_PS–cal._ = 1.10, M_n,PEG–cal._ = 1.9 kg mol^−1^, M_w,PEG–cal._ = 2.1 kg mol^−1^, Đ_PEG–cal._ = 1.09.

#### 4.4.2. Synthesis of PMeOx_20_-NH_2_

PMeOx_20_–Pht (4 g, 2.22 mmol, 1 eq) was dissolved in 100 ml EtOH and a hydrazine monohydrate solution (1 ml, 28.5 mmol, 13.5 eq) was added. The reaction mixture was heated to reflux and stirred overnight. Then, the reaction mixture was cooled to room temperature and diluted HCl_(aq.)_ was added dropwise under continuous stirring until a pH value between 2 to 3 was reached. The precipitate was filtered off. NaOH_(aq.)_ solution (1 M) was added dropwise to adjust the pH value to 7. The solution was dialysed against deionized water. The solution was freeze-dried and the polymer was obtained as a colourless solid. Yield: 2.2 g (55%). DF_NMR_ = quant., ^1^H NMR (300 MHz, CDCl_3_): *δ* [ppm] = 3.47 (br s, 66H), 2.95 (m, 4H), 2.13 (m, 60H), SEC (DMAc, 0.21 wt% LiCl, PS–cal./PEG–cal.): M_n,PS–cal._ = 4.0 kg mol^−1^, M_w,PS–cal._ = 4.5 kg mol^−1^, Đ_PS–cal._ = 1.14, M_n,PEG–cal._ = 2.0 kg mol^−1^, M_w,PEG–cal._ = 2.5 kg mol^−1^, Đ_PEG–cal._ = 1.14.

#### 4.4.3. Synthesis of PMeOx_20_-BCN

A pre-dried microwave vial was charged with PMeOx_20_-NH_2_ (254 mg, 0.14 mmol, 1 eq) dissolved in 2.5 ml distilled CHCl_3_. Then, TEA (40 µl, 0.28 mmol, 2 eq) and BCN-NHS (62 mg, 0.21 mmol, 1.5 eq) dissolved in 2.5 ml distilled CHCl_3_ were added. The reaction mixture was stirred for 23 h at room temperature in the dark under argon. The solvent was evaporated, the residue was redissolved in deionized water and dialysed. After the solution was freeze-dried, the polymer was obtained as a colourless solid. Yield: 186 mg (74%). ^1^H NMR (300 MHz, CDCl_3_): *δ* [ppm] = 4.20 (m, 2H), 3.48 (br s, 66H), 3.00 (m, 3H), 2.10 (m, 89H), 0.94 (m, 4H), SEC (DMAc, 0.21 wt% LiCl, PS–cal./PEG–cal.): M_n,PS–cal._ = 4.3 kg mol^−1^, M_w,PS–cal._ = 4.7 kg mol^−1^, Đ_PS–cal._=1.08, M_n,PEG–cal._ = 2.1 kg mol^−1^, M_w,PEG–cal._ = 2.3 kg mol^−1^, Đ_PEG–cal._ = 1.11.

#### 4.4.4. Synthesis of PMeOx_73_-Pht

A pre-heated three-necked flask with stirrer connected to a Findenser™ with T-piece and a gas bubbler was evacuated thrice, heated up and cooled under a gentle flow of argon. MeTos (379 µl, 468 mg, 2.5 mmol, 1 eq) and MeOx (17 ml, 17.1 g, 200.8 mmol, 80 eq) were dissolved in 33 ml acetonitrile, heated to reflux and stirred for 8 h under a gentle flow of argon. Subsequently, potassium phthalimide (1.2 g, 6.3 mmol, 2.5 eq) was added and the temperature was reduced to 70°C. The reaction mixture was stirred overnight, cooled to room temperature, and the precipitate was filtered off. The residue was washed with 50 ml acetonitrile, and the solvent from the combined organic layers was evaporated under reduced pressure. The crude product was dried *in vacuo*, redissolved in 100 ml deionized water, and dialysed against deionized water. The mixture was freeze-dried and the polymer was obtained as a colourless solid. Yield: 9.73 g (57%). Conversion: 97%. DP_NMR_ = 73. DF_NMR_ = 91%. ^1^H NMR (300 MHz, CD_2_Cl_2_, 300 K): *δ* [ppm] = 7.39–8.01 (m, 4H), 3.83–3.92 (m, 1H), 3.49 (br d, 294H), 3.05 (br s, 3H), 1.85–2.45 ppm (m, 280H), SEC (DMAc, 0.21 wt% LiCl, PS–cal./PEG–cal.): M_n, PS–cal_. = 12.7 kg mol^−1^, M_w,PS–cal._ = 16.8 kg mol^−1^, Đ_PS–cal._ = 1.32, M_n,PEG–cal._ = 6.8 kg mol^−1^, M_w,PEG–cal._ = 9.5 kg mol^−1^, Đ_PEG–cal._ = 1.4.

#### 4.4.5. Synthesis of PMeOx_73_-NH_2_

PMeOx_73_–Pht (8.5 g, 1.33 mmol, 1 eq) was dissolved in 51 ml EtOH, and a hydrazine monohydrate solution (0.9 ml, 18.6 mmol, 14 eq) was added. The reaction mixture was heated to 80°C and stirred overnight. Subsequently, the reaction mixture was cooled to room temperature, and diluted HCl_(aq.)_ was added dropwise under continuous stirring until a pH value of 2 was reached. The mixture was allowed to stir for 30 min before a diluted NaOH_(aq.)_ solution was added to adjust the pH value to 7. The solution was dissolved in and dialysed against deionized water. The solution was freeze-dried, and the polymer was obtained as a colourless solid. Yield: 6 g (71%). DF_NMR_ = quant., ^1^H NMR (300 MHz, CD_2_Cl_2_): *δ* [ppm] = 3.49 (br d, 294H), 3.00–3.12 (m, 3H), 2.03–2.28 (m, 216H), 1.77 (br s, 228H), SEC (DMAc, 0.21 wt% LiCl, PS–cal./PEG–cal.): M_n,PS–cal._ = 12.5 kg mol^−1^, M_w,PS–cal._ = 14.5 kg mol^−1^, Đ_PS–cal._ = 1.16, M_n,PEG–cal._ = 7.4 kg mol^−1^, M_w,PEG–cal._ = 8.8 kg mol^−1^, Đ_PEG–cal._ = 1.19.

#### 4.4.6. Synthesis of PMeOx_73_-BCN

PMeOx_73_–NH_2_ (250 mg, 0.034 mmol, 1 eq) was dissolved in 2 ml dry CH_2_Cl_2_ in a pre-dried microwave vial. Subsequently, TEA (10 µl, 0.048 mmol, 2 eq) was added via a syringe through the septum. BCN-NHS (13.8 mg, 0.047 mmol, 1.4 eq) was dissolved in 250 µl dry CH_2_Cl_2_ and added to the reaction mixture. The reaction mixture was stirred for 20 h at room temperature in the dark. The solvent was evaporated and the residue was re-dissolved in deionized water and dialysed. Afterwards, the solution was freeze-dried and the polymer was obtained as a colourless solid. Yield: 206 mg (82%). ^1^H NMR (300 MHz, CD_2_Cl_2_): *δ* [ppm] = 4.09–4.32 (m, 2H), 3.16–3.86 (m, 194H), 2.85–3.08 (m, 3H), 2.01–2.23 (m, 213H), 1.18–1.41 (m, 6H), 1.03–1.07 (m, 1H), 0.83–1.07 (m, 4H). SEC (DMAc, 0.21 wt% LiCl, PS–cal./PEG–cal.): M_n, PS–cal._ = 12.5 kg mol^−1^, M_w,PS–cal._ = 14.8 kg mol^−1^, Đ_PS–cal._ = 1.19, M_n,PEG–cal._ = 7.0 kg mol^−1^, M_w,PEG–cal._ = 8.6 kg mol^−1^, Đ_PEG–cal._ = 1.23.

### 4.5. Supporting information

Experimental information including detailed synthetic procedures for PEtOx_n_-Pht/NH_2_/BCN (*n* = 23, 80, 175), PMeOx_n_-Pht/NH_2_/BCN (*n* = 20 and 73), and additional analytical data (^1^H NMR, SEC, ESI and MALDI-TOF MS (if applicable)), summary of molar masses, dispersity indices and degree of functionalization as well as detailed information on HPLC measurements can be found in the electronic supplementary material.

## Data Availability

All data can be found in the manuscript and in the electronic supplementary material [44].
